# The Association between Food Security Status and Psychological Distress and Loneliness among Full-Time Undergraduate Students at a Minority-Serving Institution

**DOI:** 10.3390/ijerph192215245

**Published:** 2022-11-18

**Authors:** Pedro G. Guzman, James E. Lange, Amanda C. McClain

**Affiliations:** 1 School of Exercise and Nutritional Sciences, San Diego State University, San Diego, CA 92182-7251, USA; 2 Well-Being & Health Promotion Department, San Diego State University, San Diego, CA 92182-4705, USA

**Keywords:** food insecurity, psychological distress, loneliness, college students, minority-serving institution, part-time employment, full-time employment

## Abstract

Little is known about the relationship of food security (FS) status with mental health among students at minority-serving institutions. We aimed to elucidate the association of FS status with psychological distress and loneliness among full-time undergraduate students at a minority-serving institution. We used data from the National Collegiate Health Assessment III (n = 441). To assess FS, students responded to the USDA 6-item short form (range: 0–6) and responses were categorized as high (0), marginal (1) or low/very low (2–6) FS. The Kessler 6 scale assessed psychological distress (range: 0–24). The UCLA loneliness scale assessed loneliness (range: 3–9). Higher scores indicated higher psychological distress and loneliness. Using adjusted linear regression models, we examined the association of FS with psychological distress and loneliness. Compared to students with high FS (mean (SD): 9.4 (0.8)), students with marginal (11.4 (1.0); *p* < 0.05) or low/very low (11.8 (0.8); *p* < 0.01) FS had higher psychological distress scores. Compared to students with high FS (5.5 (0.3)), students with low/very low FS (6.0 (0.3); *p* < 0.05) had higher loneliness scores. Future studies should further explore these relationships using mixed methods, to provide complementary quantitative findings with the emic perspective of students and their experiences, which can inform programming to prevent and reduce food insecurity.

## 1. Introduction

Food insecurity is the lack of consistent access to healthy and safe food and is prevalent in the United States (U.S.), with specific populations experiencing disproportionately higher prevalence [1]. Notably, in the last 15 years, an increasing number of U.S. college or university students have been considered food insecure. In a recent study across 227 institutions, approximately 39% of 167,000 college students were food insecure in the last 30 days [2]. On college campuses, food insecurity appears to be more prevalent among undergraduates, especially full-time undergraduates, compared to the graduate student population [3]. Moreover, food insecurity among undergraduate college students has been shown to be associated with poor sleep quality [4], disordered eating behaviors [4], lower breakfast and home-cooked meal consumption [5], and higher stress [4] and depression [5] levels. Food insecurity among undergraduate and community college students also appears to be correlated with a lower Grade Point Average (GPA) [4,6]. These findings underscore the importance of addressing food insecurity among undergraduate college students, as poor health and academic outcomes can have greater long-term consequences to U.S. society.

Food insecurity is hypothesized to influence mental health through several pathways, which may also relate to poor college academic outcomes. Specifically, recent evidence suggests that there may be a bidirectional relationship between food insecurity and mental health. Adults with poor mental health have been shown to have a higher risk for food insecurity [7,8,9], potentially due to a limited capability to sustain employment and, thus, income [7]. Simultaneously, food insecurity has been associated with a greater number of days with poor mental health among college students, which can make the completion of tasks difficult [10], and illuminates the potential negative influence food insecurity may have on students’ health and well-being and the feasibility to function daily. Being food insecure was also previously correlated with greater feelings of loneliness among college students, suggesting that food-insecure students may be more likely to be isolated from peers, faculty, student support, staff, and family members [3]. Overall, food insecurity and mental health problems are progressively endangering the well-being of college students and impeding their success and persistence [11].

Several factors may protect or exacerbate a student’s risk of experiencing poor mental health consequences of food insecurity. For example, colleges and universities are addressing food insecurity by establishing food pantries on campus. Although food pantries are conveniently located on campus for college students, many students refuse to use food pantries because of inconvenient hours, social stigma, or not having sufficient information on the policies of food pantry use [12]. Moreover, many students working jobs part-time or full-time have experienced food insecurity because of insufficient financial resources. Even when receiving financial aid, working, and having support from their families, students previously acknowledged that lack of money and time are major contributing factors for experiencing food insecurity [13].

Considering U.S. racial/ethnic minority-headed households disproportionately experience food insecurity, four-year colleges serving diverse students may also have a higher proportion of undergraduate students at risk of food insecurity and its consequences. Before the COVID-19 pandemic, evidence indicated that food-insecure students were more likely to be younger, Black, Hispanic, low-income, employed, receiving financial aid, or housing insecure [14]. Similarly, post-COVID-19, young undergraduate students from racial/ethnic minority groups (Black, Hispanic, Asian/Pacific Islander) were more likely to experience food insecurity [14]. However, little is known about how food insecurity influences the mental health of undergraduate college student populations at minority-serving institutions such as Hispanic-Serving Institutions (HSIs) and Asian American and Native American Pacific Islander-Serving Institutions (AANAPISIs). Furthermore, identifying specific factors that moderate the food insecurity-mental health relationship among students can help inform more targeted approaches to mitigate food insecurity and its negative consequences. Thus, our study objective was to explore the association of food security status with psychological distress and loneliness among full-time undergraduate students at a minority-serving institution. In addition, we tested two moderators to these relationships—hours working for pay and use of a campus food pantry—to inform future intervention strategies.

## 2. Methods

### 2.1. Data and Sample

In the spring of 2021, approximately one year into the COVID-19 pandemic, an online survey was disseminated by the American College Health Association-National College Health Assessment (ACHA-NCHA III) to students at a public HSI and AANAPISI in California. The ACHA-NCHA is conducted annually at select U.S. universities to help discern the most common health and behavior risks affecting students’ academic performance. The purpose of the survey is to collect data about students’ health habits, behaviors, and perceptions [15]. Of the 7000 invitations sent out randomly to students to participate, a total of 687 students responded to the survey. Of these 687 students, 550 were full-time undergraduate students. The University Institutional Review Board approved the study protocols.

### 2.2. Measures

#### 2.2.1. Independent Variable: Food Security

The validated [16] United States Department of Agriculture (USDA) 6-item Food Security Survey Module short form captured food security status over the past 30 days [17]. Item questions asked about affording access to sufficient food, including balanced meals. For example, “For the following statement, please say whether the statement was often true, sometimes true, or never true for you in the last 30 days. ‘I couldn’t afford to eat balanced meals.’”. Scores were calculated by summing the number of affirmative responses to the six items (0–6). The scores were then categorized using the USDA coding scheme: high food security [zero affirmative responses], marginal [one affirmative response], low food security [2–4 affirmative responses], and very low food security [5–6 affirmative responses]. Low and very low food security statuses are often combined to represent the food insecure category and were collapsed into one category in our study to maximize sample size. High and marginal food security status are often combined to represent the food secure category, but emerging evidence, including among college students, suggests that high and marginal food security statuses are distinct and should be observed separately [18].

#### 2.2.2. Dependent Variables: Psychological Distress and Loneliness

The Kessler 6 non-specific psychological distress scale is a reliable (Cronbach’s alpha: 0.89) [19] and valid [20] instrument used to assess total estimates of moderate and serious mental health illness prevalence. The Kessler 6 scale assists with elucidating the presence of distress even if participants do not report their mental health to be poor [21]. Given that the purpose of the study was to assess psychological distress, students were asked how often in the last 30 days they felt nervous, hopeless, restless, fidgety, so sad, that everything was an effort, and worthless. For example, “For each question, please select the response that best described how often you had this feeling. ‘During the past 30 days, about how often did you feel nervous?’” Each response was given a value of zero (“none of the time”), one (“a little of the time”), two (“some of the time”), three (“most of the time”), or four (“all of the time”) [20]. The responses were summed for a score range of 0–24. Tests of normality showed score distribution in the sample was slightly right-skewed (skewness: 0.61) but with even distribution (kurtosis: −0.01).

The three-item University of California Los Angeles (UCLA) Loneliness Scale Score assessed the level of loneliness: relational connectedness, social connectedness, and self-perceived isolation [22]. For example, “Indicate how often each of the statements below is descriptive of you. ‘How often do you feel that you lack companionship?’”. The scale has shown sufficient reliability (Cronbach’s alpha: 0.62–0.88) and construct validity [23]. Response options for the three questions were one (“hardly ever”), two (“some of the time”), or three (“often”). Scores were then summed and categorized into two categories: negative for loneliness (score of 3–5) or positive for loneliness (score of 6–9) [22]. Tests of normality showed score distribution in the sample was symmetrical (skewness: 0.12) with an even distribution (kurtosis: −0.93).

### 2.3. Moderators

We tested employment status and food pantry use as moderators. The ACHA-NCHA survey asked participants how many hours they spend in a typical week working for pay. The options to respond were: 0 h, 1–5 h, 6–10 h, 11–15 h, 16–20 h, 21–25 h, 26–30 h, and more than 30 h. These options were then categorized into three distinct groups: working more than 20 h a week, working 20 h or less a week, or not currently working for pay. Students were also asked about their use of a food pantry on campus. The response options were: no, I didn’t know we had one; no I didn’t need it; yes, I have used it; and I needed it, but did not use it.

### 2.4. Covariates

We considered several covariates that could potentially be confounders in the relationship between food security status and psychological distress and loneliness, including age, sex, race/ethnicity, relationship status, being a parent or caregiver, current living situation, body mass index, alcohol use, cigarette use/smoking status, and health insurance. Race/ethnicity was assessed by how students usually described themselves: American Indian or Native Alaskan, Asian or Asian American, Black or African American, Hispanic or Latino/a/x, Middle Eastern/North African or Arab Origin, Native Hawaiian or Other Pacific Islander Native, White, Biracial or Multiracial, or another identity. Based on available sample sizes for each group, these were then categorized as non-Hispanic white, Hispanic/Latino, non-Hispanic Asian, mixed-race/ethnicity, and other. Students self-reported relationship status as not in a relationship, in a relationship/partnership (not married), or married/partnered. Parent or caregiver status was assessed by asking whether the student was a parent of a child under the age of 18 or had the primary responsibility for someone else’s children under the age of 18. Current living situation was assessed by asking students to select one of the following: campus/university housing, parent/guardian/other family member’s home, off-campus/non-university housing, temporarily with a relative/friend/couch surfing, did not have a place to live, or other. Students self-reported their height and weight. Body mass index was then calculated (kg/m^2^). Students were asked how often in the last three months they consumed beer, wine, or liquor, as well as how often in the last three months they used tobacco or nicotine (cigarettes, e-cigarettes, Juul/other vape products, water pipe or hookah, chewing tobacco or cigars). Response options included never, once or twice, monthly, weekly, and daily or almost daily. We then collapsed these responses into never, once a month or less, and more than once a month to reflect the distribution of responses. Students self-reported their primary source for health insurance: college/university health insurance plan, covered by parent/guardian plan, covered by employer-based plan, Medicaid, Medicare, State Children’s Health Insurance Program (SCHIP), Veterans Affairs/Tricare, bought a plan on their own, did not have health insurance, did not know if they had health insurance, or had insurance but did not know their primary source.

### 2.5. Analyses

We analyzed data from 441 undergraduate students with complete data. Unadjusted analyses compared participant characteristics by food security status using Chi-Square for categorical variables (Fisher’s exact test where appropriate) and ANOVA for continuous variables. To test the relationship of food security status with psychological distress and loneliness, we conducted generalized linear regression analyses, adjusting for important covariates. When modeling psychological distress, we controlled for age, sex, living situation, hours worked at a job, race/ethnicity, tobacco use, physical activity, and food pantry use. When modeling loneliness, we controlled for year in school, sex, living situation, hours worked, race/ethnicity, physical activity, and food pantry use. When comparing our dependent variables by food security status, we examined significant differences using a Tukey’s post-hoc test. We then tested two different moderation effects by including an interaction term between food security status and hours worked at a job and an interaction term between food security status and campus food pantry use. When testing campus food pantry use, we excluded those reporting “I needed it, but did not use it” because there were too few observations (n = 12) to obtain estimates. We tested each moderation effect individually for psychological distress and loneliness and used Tukey’s post-hoc test to determine significant differences. Data were analyzed using SAS 9.4 and significance was set at *p* < 0.05.

## 3. Results

### 3.1. Sample Characteristics

Among full-time undergraduate students, 50.8% were categorized with high food security, 15.4% were categorized with marginal food security, and 33.8% were categorized with low/very low food security. Students from first-generation college backgrounds or who reported current tobacco use were more likely to be categorized with low/very low food security (Table 1). Students living in off-campus non-university housing or in on-campus university housing, insured with public health insurance, reporting use of the campus food pantry, or reporting to need the campus food pantry but not using it were more likely to be categorized as marginal or low/very low food security. Students categorized with marginal or low/very low food security also tended to have higher mean psychological distress scores.

### 3.2. Food Security Status, Psychological Distress, and Loneliness

**Figure 1:**
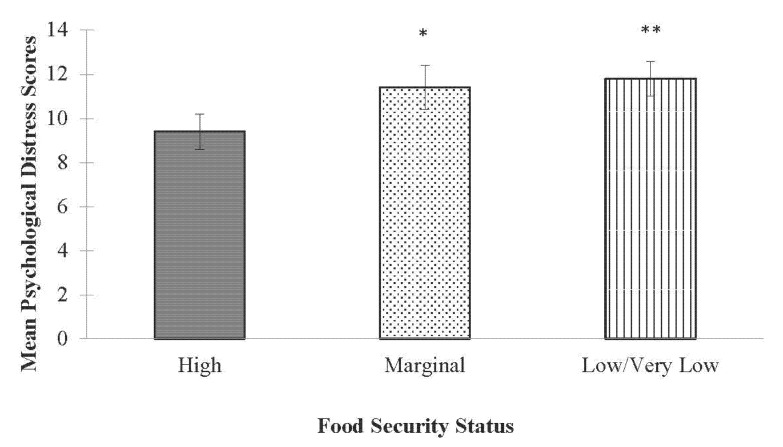
Adjusted mean (SE) psychological distress scores by food security status among full-time undergraduate students. * Indicates significantly different than high food security *p* < 0.05. ** Indicates significantly different than high food security *p* < 0.01.

**Figure 2:**
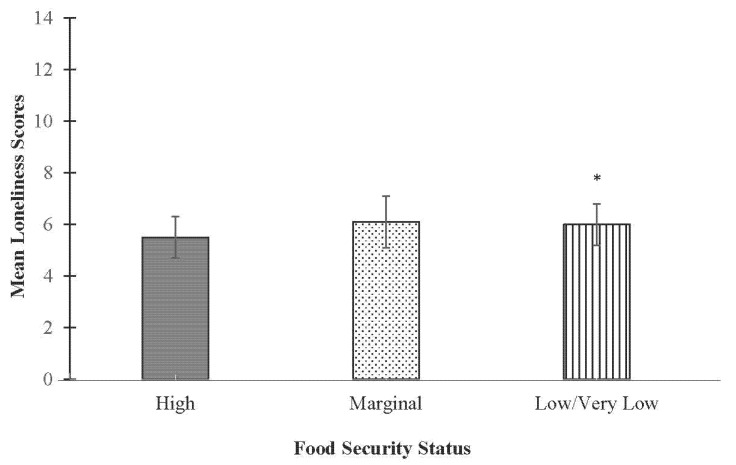
Adjusted mean (SE) loneliness scores by food security status among full-time undergraduate students. * Indicates significantly different than high food security *p* < 0.05.

In fully-adjusted models, food security status was significantly associated with psychological distress. Compared to students categorized with high food security (M (SD): 9.4 (0.8)), students categorized with marginal (11.4 (1.0); *p* < 0.05) or low/very low (10.6 (5.7); *p* < 0.01) food security had significantly higher psychological distress scores (Figure 1). In fully-adjusted models assessing the association between food security status and loneliness, food security status was significantly associated with loneliness, but only for students categorized with low/very low food security. Students in the low/very low food security group had higher loneliness scores (6.0 (0.3) *p* < 0.05) compared to students categorized with marginal (6.1 (0.3)) or high (5.5 (0.3)) food security (Figure 2).

### 3.3. Moderators

**Figure 3:**
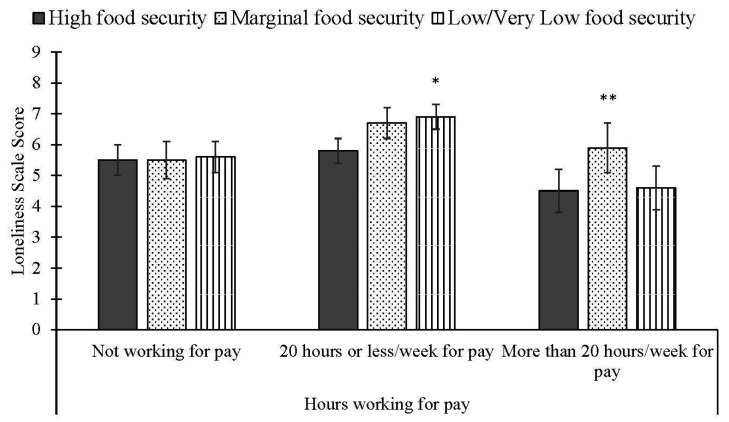
Adjusted mean (SE) loneliness scores by food security status and hours working for pay among full-time undergraduate students. * Indicates significantly different than high food security status at *p* < 0.01. ** Indicates significantly different than high food security status at *p* < 0.05; significantly different than low/very low food security status at *p* < 0.05.

Only one of our four tests of moderation effects was statistically significant. When modeling psychological distress, the interaction between food security status and hours working for pay was not significant (*p* = 0.33), nor was the interaction between food security status and food pantry use (*p* = 0.34). Similarly, for loneliness scores, the interaction of food security status and food pantry use was not significant (*p* = 0.07). However, the interaction of food security status and working for pay was significant (*p* = 0.03) when modeling loneliness scores (Figure 3). Among students reporting working 20 h a week or less for pay, loneliness scale scores were significantly higher among students with low/very low food security (6.9 (0.4); *p* < 0.01)), compared to students with high food security (5.8 (0.4)). Students reporting working 20 h a week or less for pay and categorized with marginal food security (6.7 (0.5)) did not have significantly different loneliness scores compared to students with high or low/very low food security. In addition, among students reporting working more than 20 h per week for pay, loneliness scale scores were significantly higher among students with marginal food security (5.9 (0.8); *p* < 0.05), compared to students with high (4.5 (0.7)) or low/very low (4.6 (0.7)) food security. Among students not working for pay, food security status was not significantly associated with loneliness scores (high: 5.5 (0.5); marginal: 5.5 (0.6); low/very low: 5.6 (0.5)).

## 4. Discussion

The present study sought to elucidate the associations between food security status and psychological distress and between food security status and loneliness among full-time undergraduate students at a public HSI and AANAPISI. When comparing psychological distress scores by food security status of students, psychological distress scores were significantly higher for students with marginal or low/very low food security compared to students with high food security. When comparing loneliness scores by food security status of students, loneliness scores were significantly higher for students with low/very low security compared to students with high food security, but working a job for pay appeared to moderate these relationships in nuanced ways. These results highlight the consequences to psychosocial health among full-time undergraduate students at a minority-serving institution experiencing lower levels of food security during the COVID-19 pandemic.

The present study adds to limited literature on food insecurity at minority-serving institutions. Students in our study were more likely to experience food insecurity if their parents had an educational attainment less than a bachelor’s degree, they lived off-campus, used the food pantry on campus, had public health insurance, used tobacco, or had high psychological distress. Notably, although not statistically significant, students that were third, fourth, and fifth-year students had a higher prevalence of food insecurity. These findings support past research among undergraduates from low-income families, whereby those who grew up in food-insecure homes, identified as racial/ethnic minorities, lived off-campus, or attended colleges in urban areas were more likely to report the lowest level of food security, often associated with hunger [13]. Research has shown that housing insecurity, health issues, transportation, and employment issues are prevalent predictors of food insecurity [11]. Moreover, students often rely on working part-time or full-time, Pell grants, loans, support from family, and partners to decrease their financial burden [24,25].

Our findings among undergraduate students at a public minority-serving institution in California are consistent with past literature regarding food-insecure college students experiencing more mental health distress. Among college students attending universities in the Appalachian and Southeastern Regions of the U.S., food-insecure students reported a greater number of days with poor mental and physical health, compared to food-secure students [10]. Additional research found that students experiencing high levels of stress and a depressed mood had a two-fold higher likelihood of experiencing food insecurity [26]. Feelings of being anxious, worried, or stressed have previously been reported as contributing factors affecting students’ daily life. Students described stress as affecting their academic performance because they could not concentrate, and the feeling of being angry and frustrated arose during these stressful situations [27]. Students’ well-being can be affected when they are food-insecure, thus leading to mental health challenges that can then cause students to experience higher levels of stress, anxiety, and depression [28]. Furthermore, higher levels of perceived stress and depression were significantly associated with short-term and long-term food insecurity among a diverse sample of undergraduate students at a public university in the Midwest [29]. Similar to other university student populations [30], the COVID-19 pandemic likely heightened psychological distress in our sample of undergraduate students, regardless of food security status. The pandemic significantly disrupted all aspects of university life, including limiting campus resources (e.g., housing, dining halls) and altering classroom instruction by moving it primarily to online or hybrid modes of learning, which may have negatively impacted student performance [31] and, thus, their mental health symptoms [32]. Yet, as DeBate et al. (2021) suggested, the high levels of psychological distress among students may have resulted from a combination of the pandemic and food insecurity [3]. A Fall 2020 study among more than 100,000 U.S. college and university students found that self-reported COVID-19 infections were significantly associated not only with increased odds of anxiety and depression, but also increased odds of experiencing food insecurity, demonstrating the unique challenges facing university students during this period [33].

Among undergraduate students in our study, lower levels of food security were associated with greater loneliness. The findings are consistent with a qualitative study noting that students experiencing food insecurity often felt left out or found themselves having trouble forming relationships due to a limited ability to participate in social gatherings because they either could not afford food or felt embarrassed asking friends to pay [27]. However, to the best of our knowledge, only one other study has documented the quantitative relationship between food security status and loneliness [3]. Similar to our study, the authors found that lower levels of food security were associated with higher levels of loneliness, but the study was conducted at a large university in the Southeastern U.S. and the sample included all students, not solely full-time undergraduate college students [3]. Thus, our findings contribute context-specific data for full-time undergraduate students at a minority-serving institution, which can inform future research and programming for campuses serving historically-marginalized students. The lack of research on food security status and loneliness is notable, as students who are less integrated into campus life or who are experiencing high levels of loneliness are more likely to have lower GPAs, potentially because loneliness may negatively impact student motivation to perform well in school and increase the risk of students dropping out [34]. In addition, the COVID-19 pandemic, and resulting public health measures for social distancing, may have exacerbated levels of loneliness among undergraduate students [35], emphasizing the importance of integrating social interactions with peers as a part of campus responses to promote undergraduate student well-being and success [3], especially for students from historically-marginalized backgrounds.

Our finding for the moderating role of hours working at a job are also novel. Working 20 h or less per week was associated with higher levels of loneliness among students with low/very low food security, compared to students with high food security. Although students with marginal food security did not differ significantly from students with high food security, mean loneliness scores for students with marginal food security were similar to students with low/very low food security. Notably, students with marginal food security working more than 20 h per week had significantly higher levels of loneliness, compared to students with high or low/very low food security. One potential explanation for these unexpected differences is that marginally food-secure students working 20 h a week or more exceeded income requirements to qualify for food assistance programs, such as SNAP, eliciting greater feelings of loneliness due to the social stigma associated with food insecurity, whereas, students with low/very low food security working 20 h per week or less may have not qualified for food assistance programs because they did not meet the minimum work requirements and, thus, also experienced more feelings of loneliness [36]. College students are routinely underserved by the current U.S. SNAP program and it is imperative that these government assistant programs address the unique experiences and situations of college students, including by considering the influence of their surrounding social and physical food environment on food security status [25]. Recent California legislation aims to remove barriers to SNAP participation among college students by establishing certified campus-based SNAP programs [37]. Our findings underscore the importance of widening the safety net for low-income and working-class undergraduate students to ensure their basic needs are being met for them to be successful in higher education settings.

We have several notable strengths of our study, including adding to limited studies of food insecurity at minority-serving institutions, which enroll a high percentage of students from historically-marginalized backgrounds. Our study also employed validated measures of food security, psychological distress, and loneliness. Last, we were able to control for a variety of important confounders in our analyses. Our study also has several limitations. Our study is not generalizable to other U.S. universities, especially those in other regions of the U.S. or that are not HSIs/AANAPIs. Because of the cross-sectional design of our study, we were not able to confirm the direction of the relationship between food security status and psychological distress and between food security status and loneliness. In addition, individuals with one affirmative response on the USDA 6-item short form are categorized with marginal food security, which may not be sensitive enough to distinguish them from high food security. However, the similarity of students’ psychological distress and loneliness profiles across marginal food security and low/very low food security in our study suggests the single affirmative response for marginal food security is distinct from high food security. Our sample was also primarily comprised of females, which may limit our understanding of food insecurity and mental health for other gender identities. The NCHA collects both sex and gender identity data but the small sample of participants reporting an identity other than male or female precluded us from considering differences by gender identity. Last, the quantitative design of our study limits our understanding of the lived experiences of food-insecure students and how food insecurity may manifest as psychological distress and loneliness.

## 5. Conclusions

Our findings underscore the important role that food security status plays in shaping the psychosocial health of undergraduate students on a diverse college campus, specifically a public HSI and AANAPISI, and the potential negative implications this may have on student success particularly for students from historically-marginalized backgrounds. The high prevalence of food insecurity among college students, specifically students from marginalized backgrounds and students living off-campus, has implications for university programming and future research. Our findings, in addition to previous research, underscore the need for more assistance to college students to help mitigate food insecurity, especially students who must work full- or part-time while enrolled in college. Some students have previously suggested that on-campus dining should save food and provide it to those in need instead of throwing it away, or provide at-risk students an allotted small number of meal swipes per month [38]. Last, having access to coupons or incorporating extra money for groceries, along with financial aid, are other potential interventions to assist students [38]. Universities enrolling large numbers of students from historically-marginalized backgrounds have a unique opportunity to positively shape the trajectories of health and well-being of their diverse student body as a means to promote health equity. Future studies should continue to explore the relationship of food security status with psychological distress and loneliness using mixed methods which provide complementary quantitative and qualitative findings, the latter of which can elucidate the emic perspective of students and their food insecurity and mental health experiences.

## Figures and Tables

**Table 1:** The characteristics of full-time undergraduate students by food security status (n = 441).

	Food Security Status ^1^			
	High	Marginal	Low/Very Low	
Characteristic	n = 224	n = 68	n = 149	*p*-Value
Age (y)	21.0 (3.8)	21.3 (4.5)	21.7 (3.7)	0.25
Biological sex				0.73
*Female*	75.9	79.4	74.5	
*Male*	24.1	20.6	25.5	
Highest level of parental educational attainment				0.002
*Less than a Bachelor’s degree*	32.6	36.8	51.0	
*Bachelor’s degree*	42.0	45.6	25.5	
*More than a Bachelor’s degree*	25.5	17.7	23.5	
Year in school (undergraduate level)				0.07
*1st*	23.7	20.6	13.4	
*2nd*	20.1	19.1	14.1	
*3rd*	29.9	30.9	33.6	
*4th, 5th, or more*	26.3	29.4	38.9	
Race/Ethnicity				
*Non-Hispanic White*	38.0	33.8	37.6	0.72
*Hispanic/Latino*	21.4	30.9	20.1	
*Non-Hispanic Asian*	17.9	16.2	18.1	
*Mixed Race/Ethnicity*	17.4	14.7	15.4	
*Non-Hispanic Other*	5.4	4.4	8.7	
Relationship status				0.22
*Not in a relationship*	60.3	50.0	51.0	
*In a relationship/partnership (not married)*	36.6	48.5	47.0	
*Married/partnered*	3.1	1.5	2.0	
Parent or caregiver to child(ren) under age of 18				0.41
No	94.6	97.1	97.3	
Yes	5.4	2.9	2.7	
Current living situation				0.0004
*Parent/guardian/other family member’s home*	65.2	41.2	44.3	
*Off-campus non-university housing*	26.3	45.6	41.6	
*Campus university housing*	7.1	10.3	12.8	
*Other*	1.3	2.9	1.3	
Working for pay				0.41
*No*	49.1	42.7	39.6	
*20 h or less/week*	34.4	35.3	38.9	
*More than 20 h/week*	16.5	22.1	21.5	
Use of food pantry on campus				0.0001
*No, didn’t know had one*	31.3	38.2	36.9	
*No, didn’t need it*	59.4	47.1	36.9	
*Yes, used it*	8.5	13.2	20.1	
*Needed it, but didn’t use it*	0.9	1.5	6.0	
Health insurance				0.02
*Parent/guardian plan*	80.8	72.1	68.5	
*Medicaid, Medicare, SCHIP, Veteran’s Affairs/Tricare*	8.5	11.8	17.5	
*Other plan*	8.9	7.4	8.7	
*No health insurance or don’t know*	1.8	8.8	5.4	
Body Mass Index (kg/m^2^)	23.5 (3.9)	23.8 (5.1)	24.2 (5.0)	0.31
Self-rated health				0.78
*Excellent*	11.6	8.8	10.7	
*Very good*	40.6	32.4	35.6	
*Good*	37.5	47.1	43.6	
*Fair/Poor*	10.3	11.8	10.1	
Tobacco use in last 3 months				0.03
*Never*	77.7	85.3	67.1	
*Once a month or less*	8.9	5.9	16.8	
*More than once a month*	13.4	8.8	16.1	
Alcohol use in last 3 months				0.70
*Never*	30.4	27.9	25.5	
*Once a month or less*	42.4	39.7	40.9	
*More than once a month*	27.2	32.4	33.6	
Meets weekly physical activity guidelines for aerobic and muscle-strengthening activity ^2^				0.10
*No*	55.8	69.1	54.4	
*Yes*	44.2	30.9	45.6	
Kessler non-specific psychological distress score ^3^	8.0 (4.8)	10.0 (5.8)	10.6 (5.7)	0.001
UCLA loneliness scale score ^4^	5.5 (1.8)	5.9 (2.1)	5.9 (1.8)	0.09

Data shown as either M (SD) or %, Analyses included ANOVA for continuous variables and chi-square (or Fisher exact test when appropriate) for categorical variables. SCHIP = State Children’s Health Insurance Program (SCHIP); VA = Veterans Affairs; UCLA = University of California Los Angeles. ^1^ Food security status was assessed using the USDA 6-item short form (score range: 0–6). Participants were categorized as high food security (0), marginal food security (1), or low/very low food security (2–6). ^2^ Physical activity for aerobic and muscle-strengthening encompassed how many minutes in the last seven days students spent doing moderate or vigorous physical activity or exercises to strengthen or tone muscles. ^3^ Psychological distress was assessed using the Kessler non-specific psychological distress score (score range: 0–24). Higher scores indicated greater psychological distress. ^4^ Loneliness was measured using the UCLA loneliness scale score (score range: 3–9). Higher scores indicated greater loneliness.

## Data Availability

Restrictions apply to the availability of these data. Data was obtained from San Diego State University and are available from the authors with the permission of San Diego State University.
